# Mechanisms of CO_2_ Absorption in Amino Acid-Based Deep Eutectic Solvents: Insights from Molecular Dynamics and DFT Calculations

**DOI:** 10.1021/acs.jpcb.5c00558

**Published:** 2025-05-28

**Authors:** Hung-Yi Chi, Heng-Kwong Tsao, Yu-Jane Sheng

**Affiliations:** a Department of Chemical Engineering, 33561 National Taiwan University, Taipei 106, Taiwan; b Department of Chemical and Materials Engineering, 34911 National Central University, Jhongli 320, Taiwan

## Abstract

This study explores the mechanisms of CO_2_ absorption in two amino acid-containing deep eutectic solvents (DESs) through molecular dynamics (MD) simulations and density functional theory (DFT) calculations. The MD simulations, which focus mainly on physical absorption, reveal that alanine-based DES (Ala DES) exhibits higher CO_2_ solubility than l-arginine-based DES (l-arg DES), attributed to stronger physical absorption. Furthermore, the hydrogen bond donor paired with the amino acids is identified as a critical factor for enhancing physical absorption efficiency. DFT calculations, which account for chemical absorption, investigate two reaction pathways: single-molecule reactions involving intramolecular proton transfer and two-molecule reactions involving intermolecular proton exchange. While Ala DES does not exhibit spontaneous chemical absorption, l-arg DES demonstrates such reactions, leading to the formation of carbamic acid or carbamate (Δ*G* < 0), indicative of CO_2_ capture through chemical interactions. Consequently, Ala DES primarily relies on physical absorption, whereas l-arg DES utilizes multiple reactive sites for chemical absorption. These results are consistent with experimental findings, which show that l-arg DES achieves higher CO_2_ solubility under atmospheric conditions. Overall, our study highlights the interplay between DES components and reactivity in enhancing CO_2_ capture efficiency.

## Graphical abstract

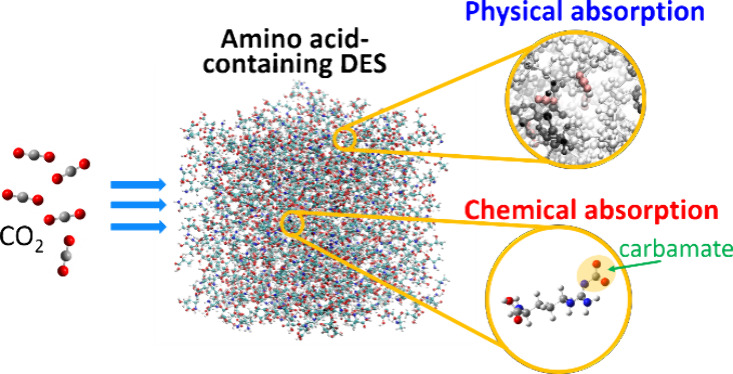

## 1 Introduction

Due to fossil fuel combustion, the soaring levels of carbon dioxide emissions have become the dominant factor in the greenhouse effect. Consequently, reducing carbon emissions has garnered significant attention and led to the development of various technologies aimed at capturing CO_2_. Solvent-based absorbents are the most widely used and established technologies for CO_2_ removal.^1–3^ Among these, aqueous amine solutions are available commercial methods for CO_2_ capture, with monoethanolamine (MEA) being the most common absorbent.^4–6^ Although aqueous amine solutions have been widely used in the petroleum industry for CO_2_ removal, they still face several challenges. The separation process of aqueous amine solutions from CO_2_ is highly energy-intensive, and issues such as corrosion and degradation remain significant concerns.^7–9^ Ionic liquids (ILs) offer advantages over aqueous amine solutions by addressing some of their shortcomings. ILs are characterized by low volatility and reduced energy requirements for the separation process.^10,11^ However, the broader application of ILs is still hindered by certain challenges. The high cost of ILs is a primary drawback, and there are also concerns about their toxicity.^12–14^

Deep eutectic solvents (DESs) can be regarded as a type of IL since at least one component is a salt. However, they address the limitations of ILs through their nontoxicity, low cost, and biodegradability, offering a more sustainable solution.^15,16^ DESs consist of two components: a hydrogen bond acceptor (HBA) and a hydrogen bond donor (HBD). The formation of hydrogen bonds between the HBA and HBD significantly lowers the melting point of DESs compared to that of either individual component.^17–21^ Based on the characteristics of HBA and HBD, DES is typically categorized into four types.^17,22^ For instance, the combination of choline chloride and urea, the most common DES, is classified as type III. Since the combination and ratio of HBA and HBD result in DESs with different properties, they serve as a potential solution for numerous fields, such as extraction,^23,24^ metal recovery,^25,26^ drug delivery^27,28^ and gas separation.^29–31^ Due to their low vapor pressure and high thermal stability,^17,23–25,29,31,32^ various DESs have been synthesized for gas separation, and some have demonstrated high CO_2_ uptake capacity, showing great potential as novel CO_2_ absorbents.^32^ Experimental results indicate that the solubility of carbon dioxide in DESs varies based on their constituent components. Additionally, operational conditions significantly affect CO_2_ capture efficiency. The CO_2_ uptake capacity of a mixture of monoethanolamine·hydrochloride (MEA·HCl) and ethylenediamine (EDA) at standard temperature and pressure has been reported.^33^ When the ratio of MEA·HCl/EDA was 1:4, an excellent CO_2_ solubility of 0.39 g CO_2_ per g DES was achieved.

The absorption capacity of an absorbent is primarily controlled by two mechanisms. Physical absorption increases CO_2_ solubility by compressing CO_2_ molecules into the absorbent under higher pressures, though this approach usually results in limited CO_2_ capture at lower pressures. Conversely, chemical absorption allows absorbents to capture CO_2_ at atmospheric pressure by incorporating CO_2_ into reaction products like carbamates.^34,35^ The amine groups in alkanolamines, such as MEA, make these molecules potentially excellent CO_2_ absorbents via chemical absorption.^36^ Similarly, amino acids also contain amine groups and are environmentally benign, suggesting they could serve as effective components in DESs for CO_2_ absorption. A DES composed of alanine (Ala) and lactic acid (Lac) showed poor CO_2_ capture ability at atmospheric pressure.^37^ However, as the pressure increased to 50 bar, this DES demonstrated a significant improvement in CO_2_ capture. In contrast, a DES consisting of l-arginine (l-arg) and varying amounts of glycerol (Gly) exhibited excellent CO_2_ solubility at atmospheric pressure.^38^

In this study, we investigate DESs containing amino acids, specifically Ala and l-arg, using molecular simulations and quantum calculations to uncover the mechanisms affecting their CO_2_ capture performance. The DES systems in this study are listed in Supporting Information Table S1 and the chemical structure of HBAs (l-arg and Ala) and HBDs (Gly and Lac) are shown in Figure 1. Our goal is to explore the roles of physical and chemical absorption in DES absorbents and offer insights for future CO_2_ capture using DESs. First, molecular dynamics (MD) simulations are employed to investigate the effect of physical absorption in DESs. The simulation results are compared with experimental data to quantify the contribution of physical absorption. Second, density functional theory (DFT), incorporating the effect of the solvent surrounding the reactants, is used to study the chemical absorption mechanisms of the amino acids. By jointly analyzing the results of MD and DFT, it is possible to determine whether physical or chemical absorption dominates CO_2_ capture in amino acid-containing DESs.

**Figure 1:**
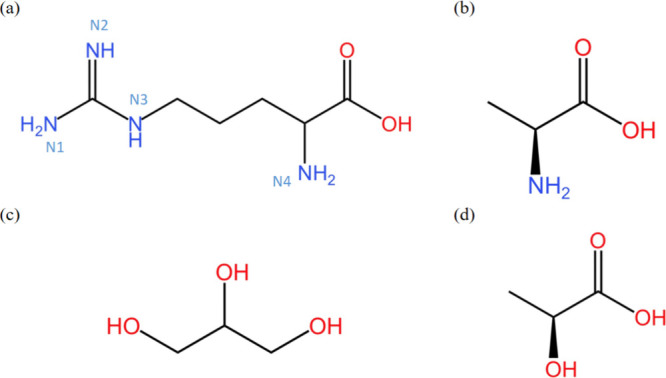
Molecular structure of (a) l-argine (l-arg), (b) alanine (Ala), (c) glycerol (Gly), and (d) lactic acid (Lac).

## 2 Methods

### 2.1 Molecular Dynamics Simulation

The liquid–vapor equilibrium was simulated using the NAMD package^39^ with the CHARMM General Force Field (CGenFF)^40,41^ for DES components and CO_2_, while water molecules were represented by the TIP4*P*/2005 rigid water model.^42^ DES components were constructed in Materials Studio 8.0 software, and their structures were optimized with the Forcite module using the COMPASS force field^43^ and an Ultra Fine quality setting. The CGenFF was subsequently employed to generate the force field and topology files for these molecules. The initial configurations of DES systems were generated by inserting HBA and HBD molecules in their respective molar ratios using Visual Molecular Dynamics (VMD),^44^ which also serves for visualization. The number of molecules and the system size in each MD simulation studied are listed in Supporting Information Table S1. In this study, temperature and pressure were controlled by the Langevin thermostat with 1 ps^–1^ damping coefficient, and Langevin barostat with 100 fs piston period and 50 fs piston decay, respectively. A time step of 1 fs was employed, and periodic boundary conditions were applied in all three dimensions for the studied systems. However, at the initial stage of the simulation, the time step is set to 0.1 fs to ensure system stability by allowing the system to gradually equilibrate and prevent numerical instabilities. Twelve angstrom cutoff radius was applied for vdW interactions and Particle Mesh Ewald method with 1.0 Å grid spacing was used for long-range interaction.

The initial DES systems underwent energy minimization to eliminate any unbalanced potential energy distribution of the molecules. The cooling process fixed at 1 atm began at 70 K above the CO_2_ capture operating temperature, with stepwise decreases of 10 K intervals until reaching the capture operating temperature. Except for the CO_2_ capture temperature, where a 2 ns NPT equilibration was performed, all other temperature steps underwent a 1 ns NPT equilibration. The final frame of the DES liquid film from NPT equilibration at the CO_2_ capture temperature was subsequently exposed to CO_2_ molecules to simulate their absorption in the DES. The equilibrated DES film was placed at the center of the simulation box along the *z*-axis, and the unequilibrated CO_2_ molecules were added into the empty space within the simulation box. We have tested CO_2_ physisorption in the Ala DES system by introducing CO_2_ before the pure DES was equilibrated. The results for the two setups are essentially the same, as expected. The absorption processes were performed in canonical ensemble (NVT) for 20 ns, ensuring the uniform distribution of CO_2_ molecules. The last 4 ns were used to analyze the distribution of CO_2_. The equilibrium configuration of absorption process is depicted in Figure 2. By adjusting the number of CO_2_ molecules and the length in the *z*-direction, different pressure values can be achieved in the absorption simulation. The number of CO_2_ and the length of simulation box in *z*-direction were presented in Supporting Information Table S2. The pressure for the absorption simulation was determined based on the gas-phase CO_2_ density after a 2 ns NVT equilibration of pure CO_2_ molecules. Note that the force field parameters used in the simulations are listed in Tables S3 and S4 of the Supporting Information.

**Figure 2:**
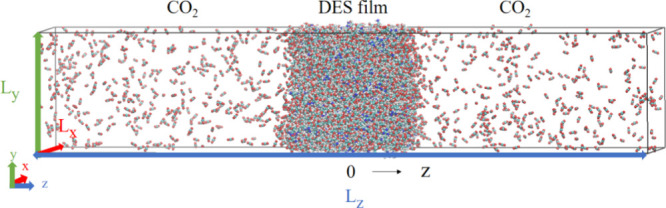
Snapshot of the equilibrium system showing liquid (DES) and vapor (CO_2_) coexistence.

### 2.2 Density Functional Theory

The DFT calculations were conducted with the Gaussian 09 software,^45^ employing Becke-Lee–Yang–Parr (B3LYP) functional^46^ and 6-311+G­(d,p) basis set. The results were visualized using GaussView 5.0.^47^ Each geometry was optimized with the continuum solvation model, solvation-model-based-on-density (SMD),^48^ to account for solvent effects. Since solvent parameters for Ala DES and l-arg DES are unavailable, and only amino acid molecules are involved in the reaction, we use the solvent parameters of HBDs available in the literature, as listed in Table 1. Missing parameters for the two HBDs are supplemented with those of other solvents having similar molecular structures to replicate the DES environment.^49^ The frequencies of all optimized structures of reactants and products were calculated at the same level to confirm true minima, ensuring no imaginary frequency. Transition state structures were optimized through TS calculations. After optimization, each structure displayed an imaginary frequency. The results were subsequently validated using intrinsic reaction coordinate (IRC).^50^ Through IRC, we calculated the structures in both forward and reverse directions to confirm consistency with the reactants and products. Both TS and IRC calculations were conducted using the same functional, basis set, and solvation model as used for the optimization of reactants and products. We have validated our DFT results using the MP2 method.^51^ The chemical reactions of Ala and l-arg with CO_2_ were tested. Compared to the DFT results based on B3LYP, there was no significant difference in structure or energy differences.

**Table 1:** SMD Parameters for HBDs^a^

solvent type	glycerol	lactic acid
Eps	42.5	19.4
epsinf	2.17	2.03
HBondAcidity	0.58*	0.60**
HBondBasicity	0.78*	0.45**
SurfaceTensionAtInterface	86.4	57.6
CarbonAromaticity	0.00	0.00
ElectronegativeHalogenicity	0.00	0.00

a * represents 1,2-ethanediol and ** stands for propanoic acid.

## 3 Results and Discussion

Two amino acid-containing DESs are considered: Ala and Lac at a ratio of 1:1, and l-arg and Gly at a ratio of 1:5, both of which have reported CO_2_ capacities from experimental studies.^37,38^ In the Section III.A, physical absorption is studied using MD to obtain the equilibrium density profile of CO_2_ in both the gas and liquid phases. After varying the system pressure, Henry’s constant can be determined. Additionally, in Section III.B, the composition of the DES is altered to understand which component of the DES predominantly affects physical absorption. Furthermore, to explore the effect of the HBD, Lac and Gly are exchanged in the two DESs to compare their CO_2_ capacities. In Section III.C, chemical absorption is investigated using DFT to determine whether the carbamate can be stably formed from amino acids. Once the carbamate is formed, the changes in enthalpy (Δ*H*) and Gibbs free energy (Δ*G*) before and after its formation are calculated. When the condition ΔG < 0 from DFT is satisfied, chemical absorption is expected to occur in DESs

### 3.1 Physical Absorption of CO_2_

The physical absorption capacity of CO_2_ can be examined through density profile analysis.^52^ The distribution of the CO_2_ molecule’s center of mass is studied along the *z*-axis, which is perpendicular to the absorbent interface. As a test, we applied this method to simulate CO_2_ dissolution in water, allowing us to confirm the approach’s effectiveness. By plotting CO_2_ concentration in water against pressure, Henry’s constant for CO_2_ in water can be determined. The density profiles of CO_2_ at different pressures are shown in the inset of Figure 3. They exhibit uniform distribution in both the gas and liquid phases. Note that sharp peaks are observed at the interface between the gas and liquid phases in each density profile in this study. The CO_2_ density at the interface could significantly inflate the physical absorption results for systems with a high interfacial area-to-volume ratio, such as our simulation box. In real systems, however, the interfacial contribution can be ignored as long as the absorbent volume is sufficiently large. The system pressure is determined by the equilibrium density of the gas phase. As shown in Figure 3, the slope of the regression line, (2.9 ± 0.2) × 10^–4^ mol/m^3^·Pa, closely matches the experimental value of 3.4 × 10^–4^ mol/m^3^·Pa,^53^ confirming that this method is suitable for studying physical absorption.

**Figure 3:**
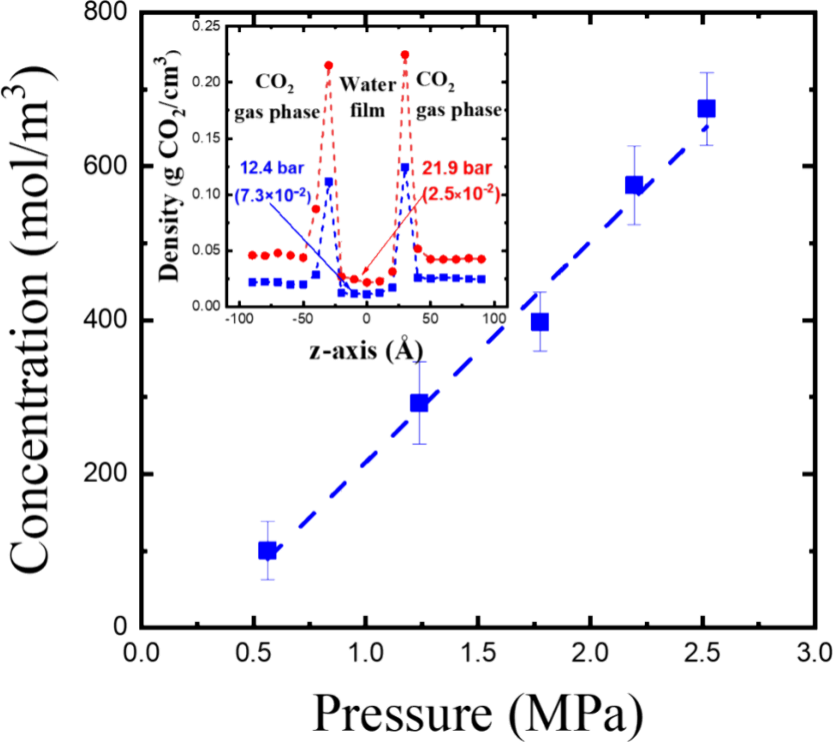
Variation of CO_2_ concentration in water with CO_2_ gas pressure. The slope represents Henry’s constant. The inset shows the density profiles of CO_2_ in the system at 12.4 and 21.9 bar. The solubility values (g CO_2_/g water) of CO_2_ in water at different pressures are indicated in the bracket in the density profile.

By replacing water with DES, the equilibrium density profiles of CO_2_ in Ala DES and l-arg DES are presented in Figure 4. Although both DESs demonstrate the ability to physically absorb CO_2_ at various pressures, they exhibit distinct performance characteristics. At similar pressures, the uniform CO_2_ concentration in Ala DES at 298 K is higher than that in l-arg DES at 333 K, which contradicts the experimental results.^37,38^ Furthermore, when comparing the distribution of CO_2_ between the DES and gas phase in each system, the CO_2_ concentration in l-arg DES is lower than in the gas phase, indicating poor CO_2_ absorption. In contrast, Ala DES exhibits a higher CO_2_ density than in its gas phase, revealing stronger physical absorption. Certainly, the CO_2_ concentration in both DESs increases as the pressure rises.

**Figure 4:**
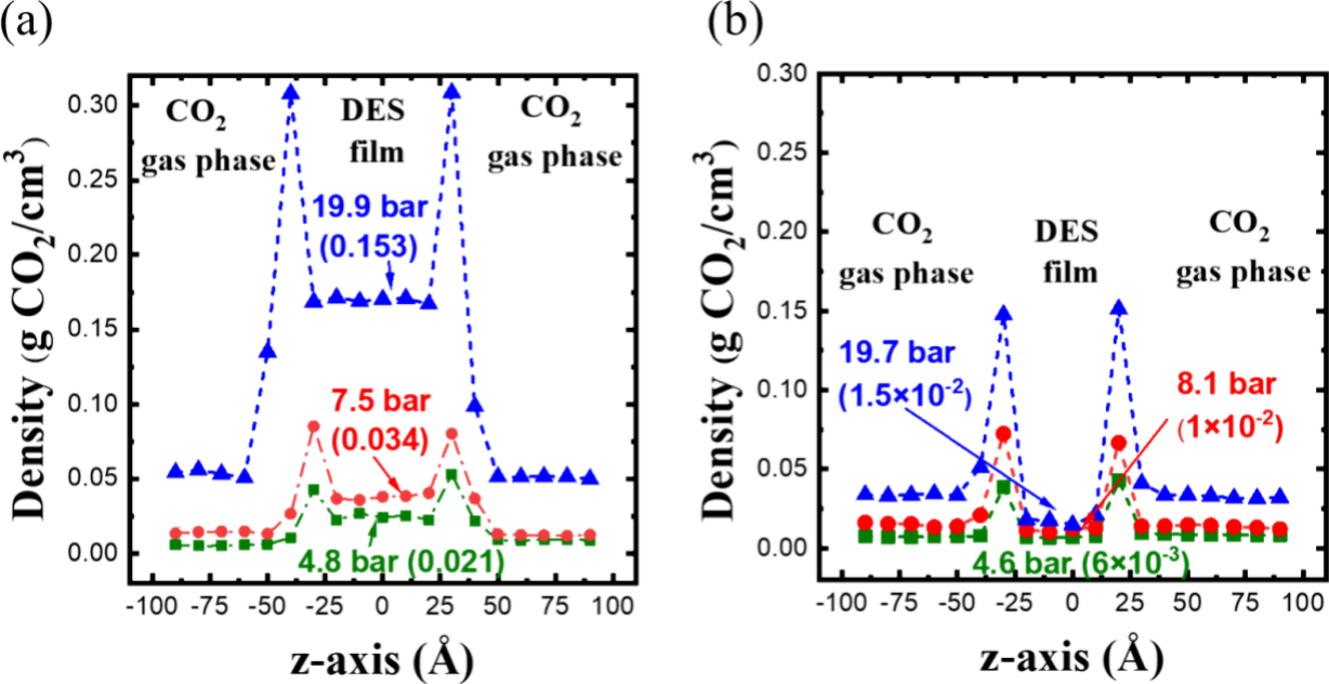
Density profiles of CO_2_ in (a) Ala DES and (b) l-arg DES at different pressures. The solubility values (g CO_2_/g DES) of CO_2_ in DES at different pressures are indicated in the bracket.

According to experimental reports, l-arg DES demonstrated significantly better performance than Ala DES. The differences between experimental results and simulation outcomes can be attributed to the absence of chemical absorption in MD simulations, which only describe physical interactions. In fact, the computational results for l-arg DES (e.g., 6 × 10^–3^ g CO_2_/g DES at 4.6 bar) are dramatically lower than the experimental value (e.g., 0.168 g CO_2_/g DES at 1 bar). Even at a pressure level of 22.7 bar, significantly higher than the experimental condition of 1 bar, the calculated value, 0.029 g CO_2_/g DES, is less than 20% of the experimental outcome, indicating that physical interactions fail to capture the absorption characteristics of l-arg DES. On the other hand, compared to the experimental results (e.g., 0.014 g CO_2_/g DES at 4.9 bar), Ala DES demonstrates similar behavior (e.g., 0.021 g CO_2_/g DES at 4.8 bar) to the experimental data, suggesting that physical interactions dominate CO_2_ capture in Ala DES.

Evidently, the CO_2_ concentration in DES changes with system pressure. Figure 5 shows the increase in CO_2_ concentration as pressure rises in the two DESs. Note that the CO_2_ concentration in DES at different pressures is calculated as the moles of CO_2_ per unit volume of DES. Since CO_2_ absorption capacity depends on pressure, it is more convenient to compare the physical capture abilities of the two DESs using Henry’s constant, represented by the slope of the regression line. Ala DES exhibits higher solubility values at all pressures, leading to a steeper slope of (2.0 ± 0.3) × 10^–3^ mol/m^3^·Pa, compared to l-arg DES with a slope of (1.9 ± 0.5) × 10^–4^ mol/m^3^·Pa. It is interesting to note that Ala DES has a Henry’s constant an order of magnitude higher than l-arg DES, which is slightly lower than that of water. We also calculated the interaction energy for the last 4 ns using the “NAMD Energy” plugin in the VMD package,^44^ as shown in Supporting Information Table S5. The nonbonded interaction energy (vdW + electrostatic) between Ala and CO_2_ is significantly stronger than that between l-arg and CO_2_. Furthermore, the radial distribution functions, presented in Supporting Information Figure S1, indicate that the interaction between the nitrogen atom in Ala and CO_2_ is stronger than that between any nitrogen atom in l-arg and CO_2_. These results clearly indicate that, in the absence of chemical absorption, Ala DES has a significantly better CO_2_ capture ability than l-arg DES due to physical interactions.

**Figure 5:**
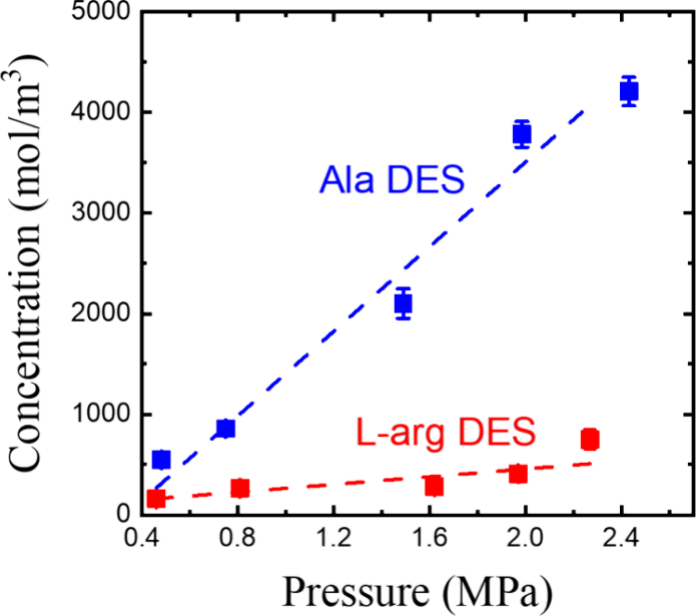
Henry’s constants of Ala DES and l-arg DES derived from the CO_2_ concentration–pressure plot.

### 3.2 Effects of DES Composition and HBD Component

So far, DESs with specific compositions have been studied for their CO_2_ absorption; however, DESs with different compositions may exhibit varying behaviors. As a result, the compositions of the two DESs are modified by adjusting the content of HBDs, Lac and Gly. This variation in composition also enables an examination of which component dominates the physical absorption mechanism. We increase the ratio of Lac in Ala DES from 1:1 to 1:2 (Ala DES*) and decrease the proportion of Gly in l-arg DES from 1:5 to 1:3 (l-arg DES*). These modified DESs are compared with the original DESs at a pressure of 12 bar. Note that, for comparison, the temperature of Ala DES* is set to match that of Ala DES, while the temperature of l-arg DES* is set to the same level as that of l-arg DES. As shown in Figure 6, the density profiles and solubility values suggest that a moderate change in composition does not lead to any noticeable improvement in physical absorption. The accumulation of CO_2_ in Ala DES* and l-arg DES* aligns with that in Ala DES and l-arg DES, respectively. The above results suggest that altering the HBD concentration does not influence the CO_2_ absorption behavior of DESs. In other words, the physical absorption capacity is not sensitive to the DES composition with moderate compositional variations.

**Figure 6:**
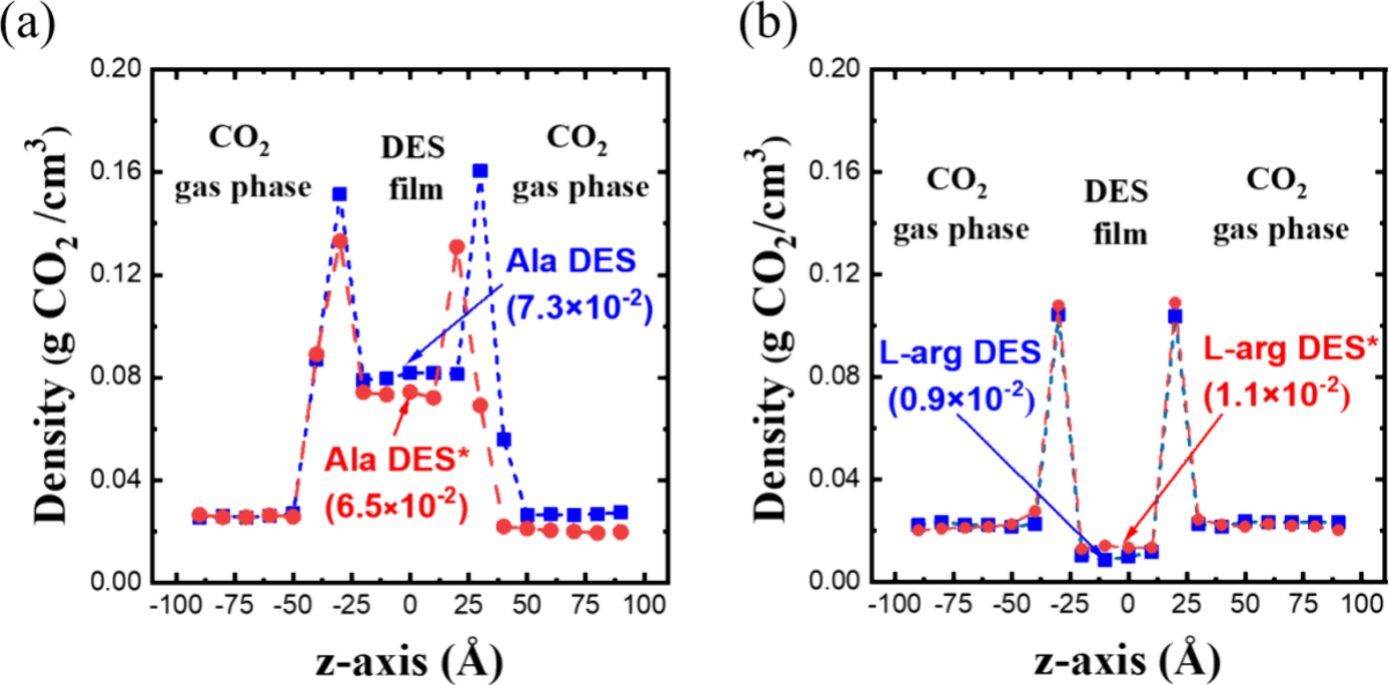
Effect of composition on CO_2_ absorption for the two DESs: (a) Ala DES and Ala DES* at 298 K and 12 bar and (b) l-arg DES and l-arg DES* at 333 K and 12 bar. The solubility values (g CO_2_/g DES) of CO_2_ in DES with different HBD concentrations are indicated in the bracket.

Aside from modifying the DES composition, the key factor driving the physical absorption mechanism can be further pinpointed by swapping the HBDs between the two DESs. Specifically, Ala is now combined with Gly (Ala/G DES), while l-arg is mixed with Lac (l-arg/L DES), both at a 1:5 ratio, and the CO_2_ capture process is assessed at 12 bar. Note that the temperatures differ between the two DESs: Ala/G DES absorbs CO_2_ molecules at 298 K, while l-arg/L DES interacts with CO_2_ molecules at 333 K. Figure 7 presents the simulation results for the two systems with exchanged HBDs, demonstrating significant changes following the HBD alteration. As shown in Figure 7a, Ala/G DES exhibits a significantly lower CO_2_ capacity (0.013 g CO_2_/g DES) compared to Ala DES (0.073 g CO_2_/g DES) when the HBD is switched from Lac to Gly. Conversely, l-arg/L DES displays a significantly higher CO_2_ capacity (0.029 g CO_2_/g DES) compared to l-arg DES (0.011 g CO_2_/g DES) when the HBD is switched from Gly to Lac. Clearly, in amino acid-containing DESs, the physical CO_2_ absorption capacity is heavily influenced by the accompanying HBD component. In fact, by swapping the HBD component, l-arg/L DES becomes to surpass Ala/G DES in terms of physical absorption. Furthermore, more CO_2_ molecules accumulate in the l-arg/L DES than in the gas phase, similar to their distribution in Ala DES. These findings suggest that the physical absorption capacity of amino acid-containing DESs is insensitive to the composition but varies significantly with the HBD component.

**Figure 7:**
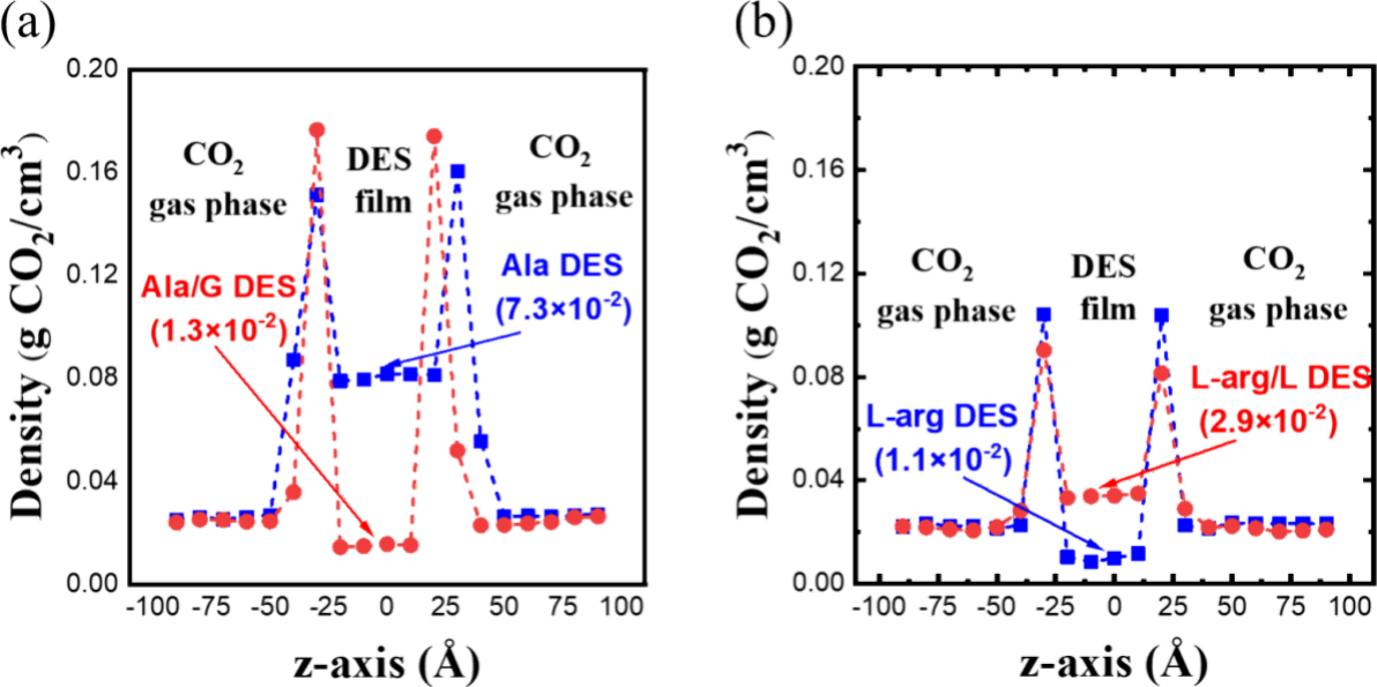
Effect of HBD components on CO_2_ absorption of amino acid-containing DESs: (a) Ala DES and Ala/G DES at 298 K and 12 bar and (b) l-arg DES and l-arg/L DES at 333 K and 12 bar. The solubility values (g CO_2_/g DES) of CO_2_ in DES with different HBDs are indicated in the bracket.

### 3.3 Chemical Absorption of CO_2_

In addition to physical absorption, CO_2_ can be chemically absorbed by DESs. The mechanism of chemical absorption involves the interaction of CO_2_ with the solvent to form a stable compound, which can later be decomposed to release the CO_2_ for storage or further use. The most widely studied and applied form of CO_2_ chemical absorption focuses on commonly used amine-based systems. The formation of carbamate is the primary chemical absorption mechanism for CO_2_ capture in amine-based solvents. The NMR spectrum reveals that carbamate forms during the CO_2_ capture process with MEA.^54^ Furthermore, previous studies using DFT calculations indicate that in the reaction between an MEA molecule and a CO_2_ molecule, carbamate forms as the primary product and is stabilized through deprotonation. Simultaneously, another MEA molecule undergoes protonation to form a quaternary amine.^55,56^ A similar mechanism can be observed with diethanolamine.^57,58^

For the chemical absorption of CO_2_ by DESs, at least one of the components must be capable of reacting with CO_2_ to form a stable compound. Due to the presence of amine groups in the amino acids within DESs, amino acid-containing DESs have significant potential for chemical absorption. If chemical interactions occur between CO_2_ and the amine groups, the simplest reaction would involve the transformation of the amine groups in amino acids into carbamate or carbamic acid. As a result, we use DFT calculations to explore the possible formation of carbamate or carbamic acid on the amine group in amino acids. To simulate the DES environment surrounding amino acid and CO_2_ reactants, we use the SMD implicit solvation model to optimize the proposed structures of both reactants and products. Unlike accurate explicit solvation models, implicit solvation models reduce computational costs while still providing excellent results.^59–61^ Compared to other implicit solvation models, such as polarizable continuum models, the SMD model requires more parameter inputs, such as surface tension and Abraham’s hydrogen bond scales, to represent different solvent types, ensuring reliable and accurate simulation of real solvents.

In our DFT calculation, first, proposed structures of the amino acid and CO_2_ molecules are optimized individually using the SMD model to calculate reactant energies. The product structure is then constructed by directly bonding the nitrogen atom of the amino acid to the carbon atom of CO_2_ to form carbamate or carbamic acid, after which it is optimized using the SMD model. Two types of absorption reactions are considered: (1) single-molecule reaction and (2) two-molecule reaction. In a single-molecule reaction, since only one amino acid participates, carbamic acid is constructed as the sole product.^55,62^ Conversely, the two-molecule reaction includes two amino acid molecules; with the presence of protonated sites, one amino acid forms carbamate, while the other undergoes protonation.^63–65^ The first type involves proton transfer within a molecule, whereas the second type involves proton transfer between two molecules.

In the Ala DES, only the Ala molecule contains an amine group that serves as a potential reactive site for CO_2_ interaction. Thus, the single-molecule reaction pathway of Ala, calculated using DFT, is illustrated in Figure 8. After optimizing the carbamic acid product structure based on ground-state electronic energy, the bond formed between the nitrogen in the amine group and the carbon in CO_2_ can stably exist. To assess the likelihood of this reaction, Δ*H* and Δ*G* must be calculated. In Figure 8a, our results indicate that Δ*H*, determined by the energy difference between the optimized structures of the product and the reactants, is negative, signifying an exothermic reaction (Δ*H* < 0). However, Δ*G* in Figure 8b, which predicts the spontaneous formation of carbamic acid, reveals that the Gibbs free energy of the product is higher than that of the reactants (Δ*G* > 0). Consequently, the formation of carbamic acid in the Ala DES is not spontaneous when the concentration effect is not taken into account. According to the equilibrium constant (K), when the reactant concentration exceeds the product concentration, product formation may still be favored even if Δ*G* > 0 (small K). As a result, when the CO_2_ concentration is very high, Ala DES may react with CO_2_ spontaneously. Notably, optimizing both the carbamate product and protonated alanine structures of two-molecule reaction yields two stable molecules. However, this reaction results in an even higher Δ*G* (39.7 kJ/mol), indicating a less favorable product. The optimized product structures of two-molecule reaction are shown in Supporting Information Figure S2.

**Figure 8:**
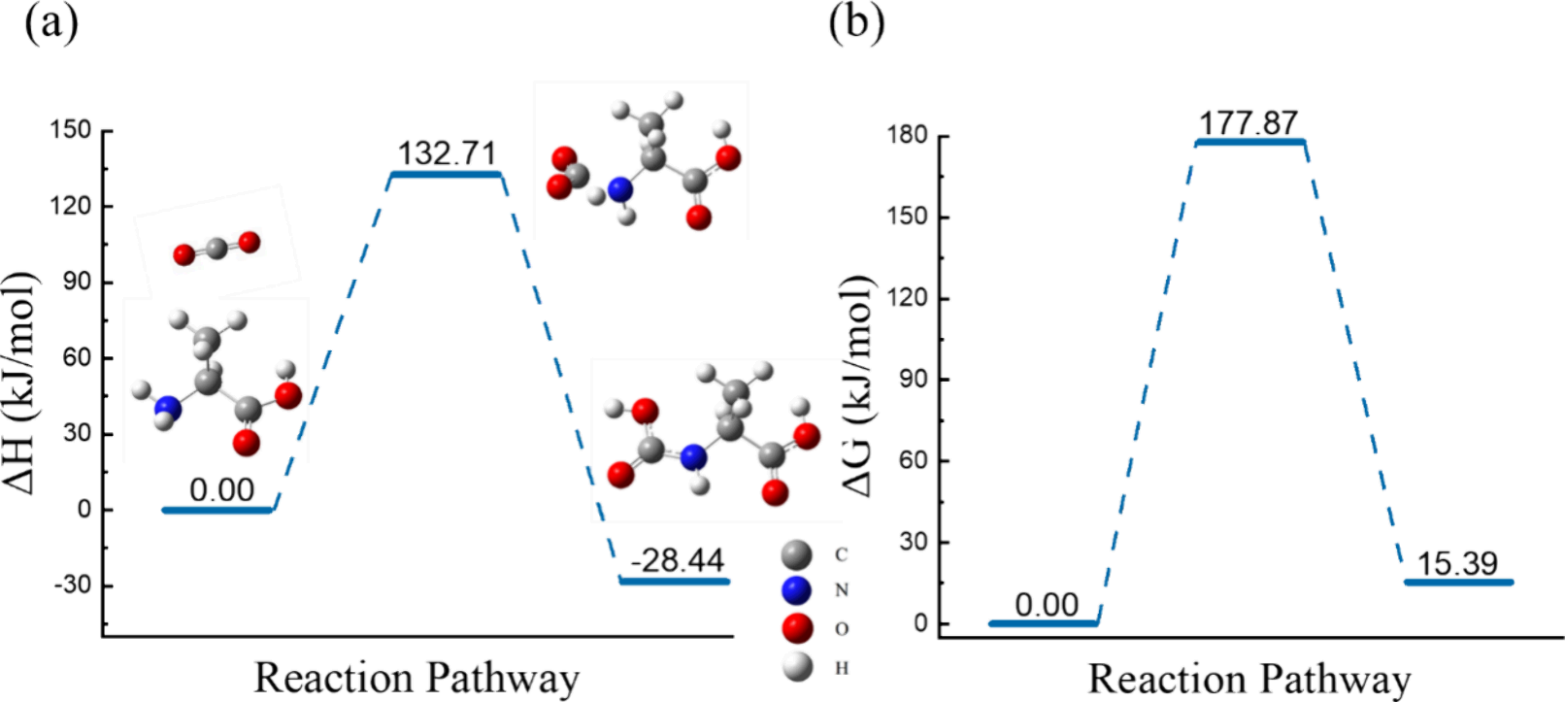
Optimized structure and (a) Δ*H* and (b) Δ*G* for the reaction pathway of CO_2_ in the Ala DES.

Unlike the Ala molecule, l-arg in the l-arg DES possesses four nitrogen atoms, each of which has the potential to serve as a reaction site for CO_2_ capture. Consequently, the formation of carbamic acid or carbamate on each nitrogen atom of l-arg is tested and shown in Figure 9. After optimizing the carbamic acid product structure, the bond formed between each nitrogen in the amine group and the carbon in CO_2_ can exist stably, as shown in Figure 9a,b,d. However, as shown in Figure 9c, the carbamic acid product structure spontaneously evolves into the carbamate product structure, with the neighboring nitrogen atom becoming protonated. Both the carbamic acid and carbamate generated on each nitrogen have a negative Δ*H*, indicating exothermic reactions. However, among all available reactive sites, only the carbamate structure associated with N3 and the protonation associated with N2, as shown in Figure 9c, exhibit a negative Δ*G*. This result indicates that l-arg can spontaneously react with CO_2_ to form a stable product. According to Figure 9, the presence of multiple nitrogen atoms allows l-arg to form carbamate through intramolecular protonation without protonating an amine group from another molecule, resulting in a stable product.

**Figure 9:**
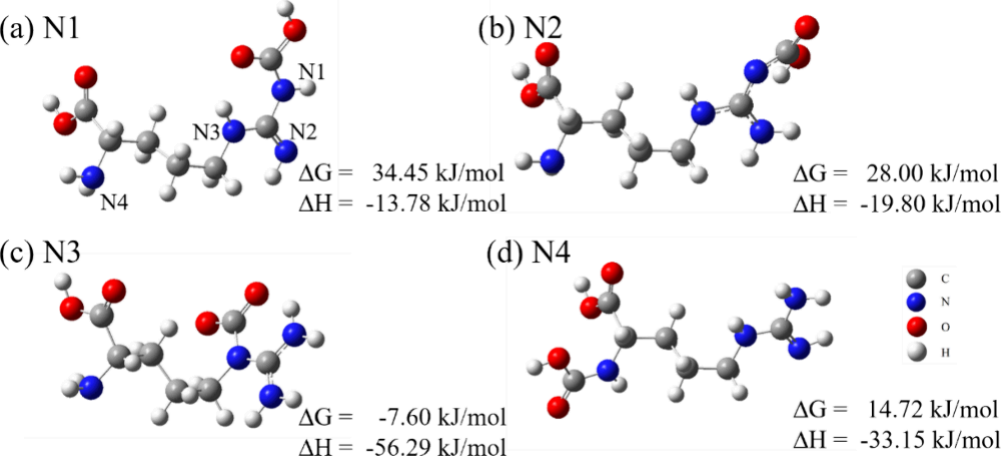
Optimized carbamic acid structures for the four nitrogen atoms of l-arg and their changes in Gibbs free energies and enthalpies. CO_2_ reacts with (a) N1, (b) N2, (c) N3, and (d) N4 on l-arg. Note that in (c), the carbamic acid structure spontaneously evolves into the carbamate structure.

In addition to the single-molecule reaction (intramolecular protonation), we also examine the two-molecule reaction of l-arg molecules, as summarized in Table 2, and present the optimized product structures in Supporting Information Figure S2. By combining carbamate formation and protonation on various nitrogen atoms of two l-arg molecules, all carbamate and protonated nitrogen species can stably coexist. Among the 16 combinations, only some result in negative Δ*H* and Δ*G*. Protonation sites at N1 and N3 do not produce negative Δ*H*, regardless of the carbamate site. When carbamate forms on the N1, N2, or N4 of one l-arg molecule, accompanied by protonation on the N2 of the other, Δ*G* becomes significantly lower than that of the single-molecule reaction. This finding indicates that, compared to the single-molecule reaction, the two-molecule reaction is a more stable and favorable CO_2_ capture mechanism, as it leads to a lower Δ*G*. Clearly, N2, which belongs to an imidoyl group and possesses only one hydrogen atom in the reactant structure, is the most reactive site for protonation. According to Table 2, carbamate formation on N3 does not occur spontaneously, regardless of the protonation site, likely because N3 is a secondary amine in the original structure of l-arg, making it less reactive. These DFT results are consistent with those from the MD simulation for RDF (Figure S1 of the Supporting Information). Figure S1b clearly demonstrates that the first peak of N3-CO_2_ is the most distant among all nitrogen atoms in l-arg.

**Table 2:** Δ*H* and Δ*G* for Chemical Absorption of CO_2_ by l-Arg Based on the Two-Molecule Reaction

carbamate site	protonated site	Δ*H* (kJ/mol)	Δ*G* (kJ/mol)	carbamate site	protonated site	Δ*H* (kJ/mol)	Δ*G* (kJ/mol)
N1	N1	40.4	87.9	N3	N1	83.4	133.3
	N2	–70.4	–26.7		N2	–27.4	18.7
	N3	31.0	73.2		N3	74.0	118.6
	N4	–14.4	33.6		N4	28.6	79.0
N2	N1	46.8	97.3	N4	N1	35.712	87.0
	N2	–63.9	–17.3		N2	–75.045	–27.6
	N3	37.5	82.6		N3	26.339	72.3
	N4	–7.90	43.0		N4	–19.066	32.7

The capture capacities of Ala and l-arg based on chemical absorption can be compared using their Δ*G*. For Ala, both single-molecule and two-molecule reactions have Δ*G* > 0. On the contrary, for l-arg, Δ*G* can be less than zero in both single-molecule and two-molecule reactions. Thus, according to DFT calculations, l-arg is a better absorbent than Ala. In contrast, based on MD simulations involving only physical absorption of CO_2_, Ala is a better absorbent than l-arg. In reported CO_2_ capture experiments, the performance of Ala is much lower, though it can be significantly improved by increasing the CO_2_ pressure. Clearly, the CO_2_ absorption of l-arg is primarily driven by chemical absorption rather than physical absorption, whereas Ala absorbs CO_2_ mainly through physical absorption rather than chemical absorption. Under low CO_2_ pressure, chemical absorption in l-arg dominates over physical absorption in Ala, explaining the remarkable experimental absorption capacity of l-arg compared to Ala at atmospheric pressure.

## 4 Conclusions

The CO_2_ absorption mechanisms of two amino acid-containing DESs are investigated using MD simulations and DFT calculations in this work. In MD simulations, which exclude chemical absorption, the Ala DES exhibits higher CO_2_ density in the liquid phase, and its solubility values under different pressures are consistent with experimental data. In contrast, l-arg DES demonstrates lower simulated solubility, indicating limited physical absorption.

To complement this, chemical absorption is assessed through DFT calculation. The results show that l-arg can react with CO_2_ to form thermodynamically favorable (Δ*G* < 0) carbamic acid and carbamate through intra- and intermolecular proton transfer, while these reactions are unfavorable for Ala. These chemical interactions enhance the CO_2_ capture capacity of l-arg DES, accounting for its exceptional reported solubility under atmospheric pressure.

In summary, the two DESs exhibit distinct CO_2_ absorption mechanisms: Ala DES captures CO_2_ purely through physical absorption, while l-arg DES captures CO_2_ mainly via chemical absorption. In addition, the role of the HBD component in enhancing physical absorption and the contribution of multiple reactive sites to chemical reactivity are examined. These findings offer valuable insights for future CO_2_ capture research.

## Supplementary Material



## Notes

Table S1: type, number of molecules, and simulation size of various bulk absorbent MD simulations, the simulation boxes for bulk DES and water are cubic, with equal lengths in all three directions; Table S2: number of CO_2_ molecules and the length of the simulation box along the *z*-axis for the solubility MD simulation under different pressures, the lengths of the simulation boxes in the *x*- and *y*-directions are set according to the results from the bulk simulations; Table S3: nonbonded force field parameters for various interaction sites in water, CO_2_, l-arg, Ala, Gly and Lac molecules; Table S4: bonded force field parameters including (a) bond, (b) angle, and (c) dihedral angle for various interaction sites in water, CO_2_, l-arg, Ala, Gly and Lac molecules; Table S5: nonbonded interaction energies of Ala-CO_2_ and l-arg-CO_2,_ total nonbonded energy is the sum of vdW energy and electrostatic energy; Figure S1: radial distribution function of (a) Ala and CO_2_ and (b) different nitrogen atoms of l-arg and CO_2_; Figure S2: optimized product structures of two-molecule reaction mechanism; carbamate formation on (a) alanine, (b) N1 of l-arg, (c) N2 of l-arg, (d) N3 of l-arg, and (e) N4 of l-arg; protonation on (f) alanine, (g) N1 of l-arg, (h) N2 of l-arg, (i) N3 of l-arg, and (j) N4 of l-arg (PDF)
