# Supplementary material for: Mechanisms of CO2 Absorption in Amino Acid-Based Deep Eutectic Solvents: Insights from Molecular Dynamics and DFT Calculations
Source: J Phys Chem B. 2025 May 28;129(23):5779–87. doi: 10.1021/acs.jpcb.5c00558 (PMC12169667; doi:10.1021/acs.jpcb.5c00558)
Supplement: Supplementary file 1 [file jp5c00558_si_001.pdf]

Mechanisms of CO_2 Absorption in Amino Acid-Based Deep Eutectic Solvents: Insights from Molecular Dynamics and DFT Calculations

Hung-Yi Chi[a], Heng-Kwong Tsao[b,*] and Yu-Jane Sheng[a,*]

[a]Department of Chemical Engineering, National Taiwan University, Taipei 106, Taiwan
[b]Department of Chemical and Materials Engineering, National Central University, Jhongli 320, Taiwan

Corresponding Authors

*E-mail: hktsao@cc.ncu.edu.tw (H.-K.T.); yjsheng@ntu.edu.tw (Y.-J.S.).

*Tel.: +886 3 422 7151#34225 (H.-K.T.); +886 2 33663014 (Y.-J.S.).

Supplementary Information

Table S1. The type, number of molecules, and simulation size of various bulk absorbent MD simulations. The simulation boxes for bulk DES and water are cubic, with equal lengths in all three directions.

MD System	Type of Component	Number of Molecules	System Size (Å)
Ala DES	Ala/Lac	900/900	62.2
Ala DES*	Ala/Lac	600/1200	62.1
Ala/G DES	Ala/Gly	270/1350	59.5
L-arg DES	L-arg/Gly	200/1000	56.8
L-arg DES*	L-arg/Gly	290/870	56.8
L-arg/L DES	L-arg/Lac	230/1150	59.5
Water	water	6000	56.6

Table S2. The number of CO_2 molecules and the length of the simulation box along the z-axis for the solubility MD simulation under different pressures. The lengths of the simulation boxes in the x- and y-directions are set according to the results from the bulk simulations.

MD System	Pressure (MPa)	Number of CO_2	Length in z-direction (Å)
Water	0.56	500	920.0
Water	1.24	1000	920.0
Water	1.78	1000	640.0
Water	2.19	1000	471.0
Water	2.52	1000	400.0
Ala DES	0.48	500	962.0
Ala DES	0.75	500	470.0
Ala DES	1.26	1000	462.0
Ala DES	1.49	1000	390.0
Ala DES	1.99	2000	462.0
Ala DES	24.3	2000	360.0
L-arg DES	0.48	250	540.0
L-arg DES	0.81	250	260.0
L-arg DES	1.28	500	360.0
L-arg DES	1.62	500	300.0
L-arg DES	1.97	1000	490.0
L-arg DES	2.27	1000	390.0
Ala DES*	1.21	1000	462.0
L-arg DES*	1.27	1000	770.0
Ala/G DES	1.28	1000	570.0
L-arg/L DES	1.27	1000	680.0

Table S3. Non-bonded force field parameters for various interaction sites in water, CO_2, L-arg, Ala, Gly and Lac molecules.

Compound	Atom	R_{min} (Å)	ε (kcal/mol)	q (e)
Water (TIP4P/2005)	O	3.5452	0.1852	0
	H	0	0	+0.5564
	M	0	0	-1.1128
CO_2	C	3.1260	0.0580	0.6860
	O	3.3840	0.1650	-0.3430
L-arg	C (C-N_3)	4.0000	0.1100	0.5460
	N (NH_2)	3.7000	0.2000	-0.9120
	N (N=C)	2.9800	0.0600	-0.5990
	N (C-NH)	4.0000	0.0450	-0.5290
	C (N-CH_2)	4.0200	0.0560	-0.0340
	C (CH_2)	4.0200	0.0560	-0.2130
	C (CH_2)	4.0200	0.0560	-0.2870
	C (CH-N)	4.0000	0.0320	0.3260
	C (COOH)	3.4000	0.0980	0.7790
	O (C=O)	3.4000	0.0120	-0.5480
	O (C-OH)	3.5300	0.1921	-0.5530
	N (HC-N_2)	3.9800	0.0600	-1.0770
	H (C=NH)	0.4490	0.0460	0.3700
	H (NH_2)	1.6500	0.0100	0.3300
	H (NH_2)	1.6500	0.0100	0.3300
	H (C-NH)	2.6800	0.0350	0.3590
	H (CH_2)	2.6800	0.0350	0.0900
	H (CH_2)	2.6800	0.0350	0.0900
	H (CH_2)	2.6800	0.0350	0.0900
	H (CH_2)	2.6800	0.0350	0.0900
	H (CH_2)	2.6800	0.0350	0.0900
	H (CH_2)	2.6800	0.0350	0.0900
	H (H-C-N)	2.6800	0.0450	0.0900
	H (C-OH)	0.4490	0.0460	0.2900
	H (HC-NH_2)	1.6500	0.0100	0.3960
	H (HC-NH_2)	1.6500	0.0100	0.3960
Ala	C (C-H)	4.0000	0.0320	0.2130
	C (C-OOH)	3.4000	0.0980	0.7940
	C (C-H_3)	4.1000	0.0780	-0.2820

	N	3.9800	0.0060	-1.0650
	O (O=C)	3.4000	0.0120	-0.5490
	O (O-H)	3.5300	0.1921	-0.5530
	H (H-C)	2.6800	0.0450	0.0900
	H (H_3-C)	2.6800	0.0240	0.0900
	H (H_3-C)	2.6800	0.0240	0.0900
	H (H_3-C)	2.6800	0.0240	0.0900
	H (H_2-N)	1.7500	0.0010	0.3960
	H (H_2-N)	1.7500	0.0010	0.3960
	H (H-O)	0.4490	0.0460	0.2900
Gly	C (CH_2-OH)	4.0200	0.5600	0.0450
	C (CH-OH)	4.0000	0.032	0.1490
	C (CH_2-OH)	4.0200	0.5600	0.0450
	O	3.5300	0.1921	-0.6500
	O	3.5300	0.1921	-0.6470
	O	3.5300	0.1921	-0.6500
	H (CH_2)	2.6800	0.0350	0.0900
	H (CH_2)	2.6800	0.0350	0.0900
	H (CH)	2.6800	0.0450	0.0900
	H (CH_2)	2.6800	0.0350	0.0900
	H (CH_2)	2.6800	0.0350	0.0900
	H (OH)	2.6800	0.0450	0.4190
	H (OH)	2.6800	0.0450	0.4200
	H (OH)	2.6800	0.0450	0.4190
Lac	C (CH_3)	4.1000	0.0780	-0.2810
	C	4.0000	0.0320	0.1750
	C (COOH)	3.4000	0.0980	0.7850
	O (O=COH)	3.4000	0.1200	-0.5650
	O (OC-OH)	3.5300	0.1921	-0.5210
	O (CH-OH)	3.5300	0.1921	-0.6580
	H (CH_3)	2.6800	0.0240	0.0900
	H (CH_3)	2.6800	0.0240	0.0900
	H (CH_3)	2.6800	0.0240	0.0900
	H (CH)	2.6800	0.0450	0.0900
	H (COOH)	0.4490	0.0460	0.2900
	H (CH-OH)	0.4490	0.0460	0.4150

The functional form of non-bonded force field:

- Electrostatic interaction

$$\frac{q_i q_j}{\varepsilon\, r_{ij}}$$

- Van der Waals interaction

$$\sum_{nonbonded} \varepsilon[(\frac{R_{\min ij}}{r_{ij}})^{12} - (\frac{R_{\min ij}}{r_{ij}})^{6}]$$

r_{ij} = distance of atom pair

$R_{\min ij}$ = where the Lennard-Jones potential is zero

Table S4. Bonded force field parameters including (a) bond, (b) angle, and (c) dihedral angle for various interaction sites in water, CO_2, L-arg, Ala, Gly and Lac molecules.

(a) Bond<br>Compound	Type	K_b (kcal/mol)	r_0 (Å)
Water (TIP4P/2005)	O-H	450.00	0.9572
	O-M	0.00	0.1500
CO_2	C=O	986.00	1.1700
L-arg	C=N	500.00	1.3100
	C-N (C-NH_2)	450.00	1.4400
	C-N (C-NH)	500.00	1.4400
	N-H (C=NH)	455.00	1.0000
	N-H (N=C-NH)	453.10	1.0140
	N-C (N-C-C)	263.00	1.4740
	N-H (C-NH-C)	447.80	1.0190
	C-C (N-C-C)	222.50	1.5300
	C-H (N-CH_2)	309.00	1.1110
	C-H (CH_2-CH_2)	309.00	1.1110
	C-C (C-C)	222.50	1.5300
	C-C (C-C-NH)	222.50	1.5380
	C-C (C-COOH)	200.00	1.5220
	C-N (HC-NH_2)	263.00	1.4740
	C-H (HC-NH_2)	309.00	1.1110
	C=O	750.00	1.2200
	C-O	230.00	1.440
	O-H	545.00	0.9600
	N-H (CH-N-H_2)	453.10	1.0100
Ala	C-C (NC-COOH)	200.00	1.522
	C-C (NC-CH_3)	222.50	1.5830
	C-N(C-HC_2O)	263.00	1.4740
	C-H (NC-H)	309.00	1.1110
	C=O	750.00	1.2200
	C-O	230.00	1.4000
	C-H (C-H_3)	322.00	1.1110
	C-H (C-H_3)	322.00	1.1110
	C-H (C-H_3)	322.00	1.1110
	N-H	453.10	1.0140
	N-H	453.10	1.0140

	O-H	545.00	0.9600
Gly	C-C (CH-CH_2)	222.50	1.3800
	C-O	428.00	1.4200
	C-H (CH_2)	309.00	1.1110
	C-H (CH)	309.00	1.1110
	O-H	545.00	0.9600
Lac	C-C (CH_3-C)	222.5	1.5380
	C-H (CH_3)	322.0	1.1110
	C-C (C-COOH)	200.0	1.5220
	C-O (CH-OH)	230.0	1.4000
	C-H (OC-H)	309.0	1.1110
	C=O	750.0	1.2200
	C-O (CO-OH)	230.0	1.400
	O-H (COOH)	545.0	0.960
	O-H (CH-OH)	545.0	0.960
(b) Angle			
Compound	Type	K_θ (kcal/mol)	θ_0 (degrees)
Water (TIP4P/2005)	H-O-H	55.00	104.52
CO_2	O=C=O	45.00	180.00
L-arg	C-N-H (N_2C-N-H)	45.00	106.00
	C-N-C(N_2C-N- CH_2)	43.00	106.00
	C-N-H (N_2C-N-H_3)	55.00	108.00
	N-C-N(N=C-NH)	50.00	125.00
	N-C-N(N=C-NH_3)	100.0	125.00
	N-C-N(NH_3-C-NH)	50.00	113.00
	N-C-H (NH-C-H)	32.40	109.50
	N-C-C(NH-CH_2-CH_2)	43.70	110.00
	C-C-H(CH_2-CH-H)	26.50	110.10
	C-C-C(N_3CH_2-CH_2-CH_2)	58.35	113.60
	C-N-H(CH_2-NC-H)	35.00	111.00
	C-C-C (CH_2-CH_2-CN)	58.35	113.50
	C-C-H (CH_2- CNH_2-H)	34.50	110.10
	C-C-N (CH_2- CH-NH_2)	43.70	112.20

	C-C-C (C- C-COOH)	52.00	108.00
	C-N-H (HC-N-H)	41.00	112.10
	C-C-O (C-C-COH)	55.00	110.50
	C-C=O	70.00	125.00
	C-C-H (NC-C-H)	33.43	110.10
	C-O-H	55.00	115.00
	C-C-H(COOH-C-H)	50.00	109.50
	C-C-N(COOH-C-N)	43.7	110.00
	O=C-O	50.00	123.00
	N-C-H (NH_2-C-H)	32.40	109.50
	H-N-H	42.00	105.85
	H-C-H	35.30	109.00
Ala	C-N-H	41.00	112.10
	C-C-H (NC-C-H)	37.50	110.10
	C-C-O	55.00	110.50
	C-C=O	70.00	125.50
	C-O-H	55.00	115.0
	C-C-H (H-C-C-O)	50.00	109.50
	C-C-N (O=C-C-N)	43.70	110.0
	C-C-C	52.00	108.00
	C-C-H (C-CN-H)	34.5	110.10
	C-C-H (CH_3-C-N)	73.00	113.20
	N-C-H	32.40	109.50
	O-C-O	50.00	123.00
	H-C-H	35.50	108.40
	H-N-H	42.00	105.85
Gly	C-O-H (CH_2)	50.00	106.00
	C-C-H (CH_2-C-H)	34.50	110.10
	C-C-O (CH_2-C-O)	75.70	110.00
	C-C-C	58.35	113.50
	C-O-H (CH)	50.00	106.00
	C-C-H (CH-CH-H)	33.40	110.10
	C-C-O (C-CH_2-O)	33.43	110.10
	O-C-H	45.90	108.90
	H-C-H	35.50	109.00
Lac	C-C-H (CH_3-C-H)	34.50	110.10
	C-C-O (CH_3-C-O)	75.70	110.10

	Type			
	C-C-C	52.00	108.00	
	C-O-H (CH-O-H)	50.00	106.00	
	C-C-O (CH-COH)	55.00	110.50	
	C-C=O	70.00	125.00	
	C-C-H (C-CH_3)	33.43	110.10	
	C-O-H (COOH)	55.00	115.00	
	C-C-H (COOH-C)	50.00	109.50	
	C-C-O (COOH-C)	110.00	106.70	
	O-C=O	50.00	123.00	
	O-C-H (CH)	45.90	108.89	
	H-C-H	35.50	108.40	

(c) Dihedral angle

Compound	Type	K_χ (kcal/mol)	n (multiplicity)	δ (degrees)
L-arg	C-N-C-C	0.800	1	180.00
	N=C-N-H (C-N-C)	2.800	2	180.00
	N=C-N-C	0.500	2	180.00
	N=C-N-H(NH_2)	0.200	2	180.00
	N-C-N-H (NH_2-C-N-H)	2.80	2	180.00
	N-C=N-H(NH_2)	5.200	2	0180.00
	N-C-C-H	0.195	3	0.00
	N-C-C-C (NH)	0.300	3	0.00
	N-C-N-H(NH-C-NH-H)	0.200	2	180.00
	N-C=N-H (NH_2)	5.200	2	180.00
	C-C-C-H (CH_2-CH_2- CH-H)	0.195	3	0.00
	C-C-C-C(CH_2- CH_2-CH_2- CHN)	0.500	6	180.00
	C-C-C-H (CH_2-CH_2- CN-H)	0.195	3	180.00
	C-C-C-N(CH_2- CH_2-CH-NH_2)	0.195	3	0.00
	C-C-N-H (CH_2- CH-NH-H)	0.400	3	0.00
	C-C-C-O (OH)	0.500	6	180.00
	C-C-C=O	0.500	6	180.00
	C-C-O-H	2.050	2	180.00

	C-C-C-H (NC-CH_2-CH-H)	0.195	3	1.00
	C-C-N-H(COOH)	0.160	3	0.00
	C-C-C-H (COOH)	0.200	3	0.00
	O=C-O-H	2.050	2	180.00
	O=C-C-H	0.00	3	0.00
	O=C-C-N	0.00	6	180.00
	O-C-C-H	0.050	6	180.00
	O-C-C-N	0.00	6	180.00
	N-C-C-H (NH_2)	0.160	3	0.00
	H-C-C-H (CH_2-CH_2)	0.220	3	0.00
	H-N-C-H (NH- CH_2)	0.500	3	0.00
	H-C-C-H (CH_2-CNH_2-H)	0.195	3	0.00
	H-C-N-H (H-C-NH-H)	0.100	3	0.00
	C-N-C-H	0.00	3	180.00
	C-N-C-C	0.800	1	180.00
	N-C-N-C	0.500	2	180.00
	C-C-C-C (CH_2- CH_2-CNH-COOH)	0.200	3	0.00
	C-C-N-H (CH_2-CH_2-N-H)	0.300	3	0.00
Ala	C-C-O-H	2.050	2	180.00
	C-C-N-H	0.160	3	0.00
	C-C-C-H	0.200	3	0.00
	C-C-N-H	0.500	1	0.00
	N-C-C-H	0.160	3	0.00
	O-C-C-N	0.000	1	0.00
	O-C-C-C	0.050	6	180.00
	O-C-O-H	2.050	2	180.00
	O-C-C-H	0.000	3	0.00
	O=C-C-H	0.050	6	180.00
	H-C-N-H	0.010	3	0.00
	H-C-C-H	0.195	3	0.00
Gly	C-C-O-H(CH_2-CH)	0.300	1	0.00
	C-C-C-H	0.195	2	0.00

	C-C-C-O	0.200	3	180.00
	C-C-O-H(CH-CH_2)	1.130	1	0.00
	O-C-C-H(CH_2-CH)	0.195	3	0.00
	O-C-C-O	0.200	3	0.00
	O-C-C-H(CH_2-CH)	0.195	3	180.00
	H-C-O-H	0.180	3	0.00
	H-C-C-H	0.195	3	0.00
	H-C-O-H	0.180	3	0.00
Lac	C-C-O-H(CH_3-CH)	1.330	1	0.00
	C-C-C=O	0.050	6	180.00
	C-C-O-H (COH-COOH)	2.050	2	180.00
	C-C-O-H (COOH-COH)	0.085	1	180.00
	C-C-C-H	0.200	3	0.00
	O=C-O-H	2.050	2	180.00
	O=C-C-H	0.000	3	0.00
	O-C-C-O	0.050	6	180.00
	O-C-C-H (C-CH_3)	0.200	3	0.00
	H-C-C-H	0.1950	3	0.00
	H-C-O-H	0.00	3	0.00
	O=C-C-O	0.050	6	180.00
	C-C-C-O	0.050	6	180.00

The functional form of non-bonded force field:

- Bonded potential energy:

$$\sum_{bonded} K_b\,(r - r_0)^2$$

- Angular potential energy:

$$\sum_{angle} K_\theta\,(\theta - \theta_0)^2$$

- Dihedral potential energy:

$$\sum_{dihedral} K_\chi\,(1 + \cos(n\psi + \delta))$$

Table S5. The nonbonded interaction energies of Ala-CO_2 and L-arg-CO_2. Total nonbonded energy is the sum of vdW energy and electrostatic energy.

Pairs	vdW (kcal/mol)	Electrostatic (kcal/mol)	Total (kcal/mol)
Ala-CO_2	-410.5 ± 13.2	-730.0 ± 23.8	-1140.5 ± 36.8
L-arg-CO_2	-52.8 ± 3.2	-109.4 ± 5.2	-162.2 ± 8.1

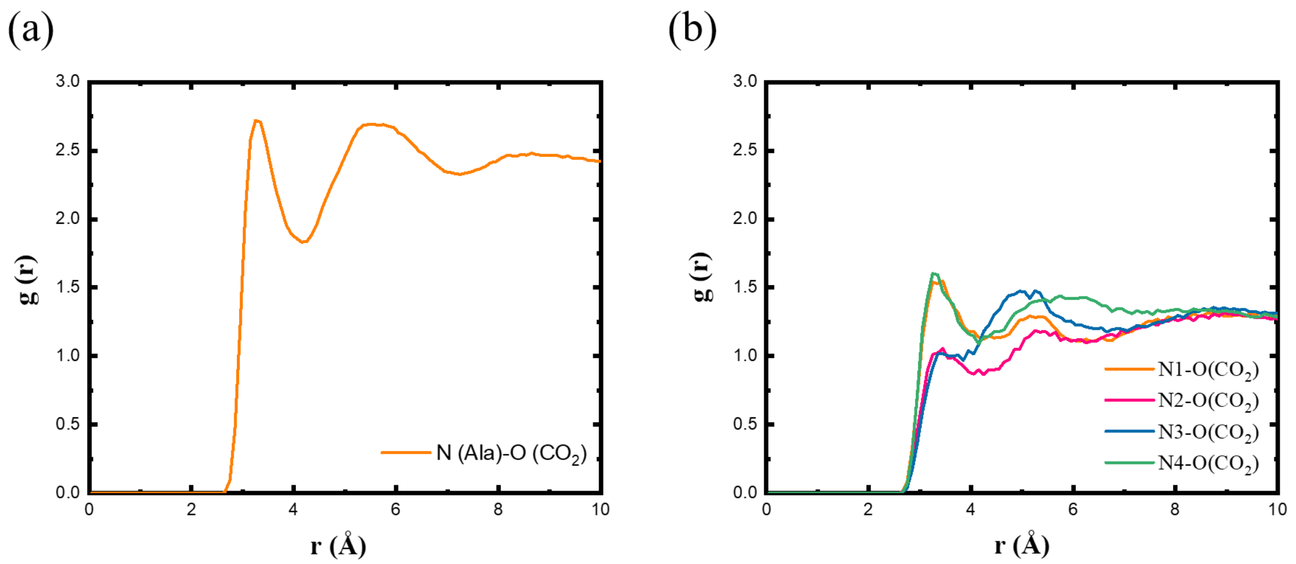

Figure S1. Radial distribution function of (a) Ala and CO_2, and (b) different nitrogen atoms of L-arg and CO_2.

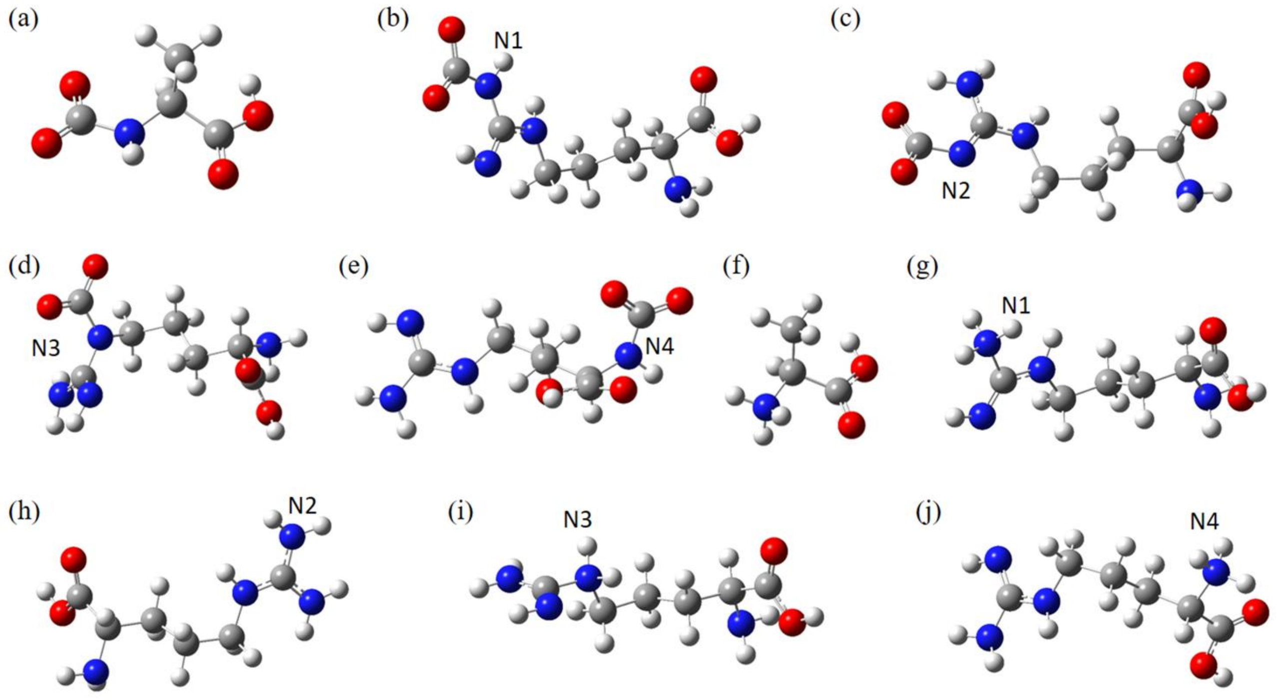

Figure S2. Optimized product structures of two-molecule reaction mechanism. Carbamate formation on (a) alanine, (b) N1 of L-arg, (c) N2 of L-arg, (d) N3 of L-arg, and (e) N4 of L-arg. Protonation on (f) alanine, (g) N1 of L-arg, (h) N2 of L-arg, (i) N3 of L-arg, and (j) N4 of L-arg.
